# An Incidental Diagnosis of SARS-CoV-2 Pneumonia With Magnetic Resonance Imaging

**DOI:** 10.7759/cureus.12115

**Published:** 2020-12-16

**Authors:** Marco Di Girolamo, Emanuele Muscogiuri, Alberto Zucchelli, Andrea Laghi

**Affiliations:** 1 Department of Radiology, Sant'Andrea Hospital - Sapienza University of Rome, Roma, ITA

**Keywords:** sars-cov-2, interstitial pneumonia, sars-cov-2 related pneumonia, magnetic resonance imaging, covid-19 pneumonia

## Abstract

The Coronavirus disease 2019 (COVID-19) is caused by the human severe acute respiratory syndrome coronavirus-2 (SARS-CoV-2) virus. The most common clinical findings related to COVID-19 are fever and cough, with the proportion of patients developing interstitial pneumonia. Other symptoms include dyspnea, expectoration, headache, anosmia, ageusia, myalgia and malaise. To date, the diagnostic criteria for COVID-19 include nasopharyngeal and oropharyngeal swabs. Computed tomography (CT) scans of the thorax showing signs of interstitial pneumonia are important in the management of respiratory disease and in the evaluation of lung involvement. In the literature, there are few cases of COVID-19 pneumonia diagnosis made using magnetic resonance imaging (MRI). In our report, we describe a case of accidental detection of findings related to interstitial pneumonia in a patient who underwent abdominal MRI for other clinical reasons. A 71-year-old woman was referred to our department for an MRI scan of the abdomen as her oncological follow-up. She was asymptomatic at the time of the examination and had passed the triage carried out on all the patients prior to diagnostic tests during the COVID-19 pandemic. The images acquired in the upper abdomen showed the presence of areas of altered signal intensity involving asymmetrically both pulmonary lower lobes, with a patchy appearance and a preferential peripheral subpleural distribution. We considered these features as highly suspicious for COVID-19 pneumonia. The nasopharyngeal swab later confirmed the diagnosis of SARS-CoV-2 infection. There are limited reports about MRI features of COVID-19 pneumonia, considering that high-resolution chest CT is the imaging technique of choice to diagnose pneumonia. Nevertheless, this clinical case confirmed that it is possible to detect MRI signs suggestive of COVID-19 pneumonia. The imaging features described could help in the evaluation of the lung parenchyma to assess the presence of signs suggestive of COVID-19 pneumonia, especially in asymptomatic patients during the pandemic phase of the disease.

## Introduction

In late December 2019, the newly described severe acute respiratory syndrome coronavirus-2 (SARS-CoV-2) was reported as the etiologic agent of COVID-19 pneumonia [1,2]. Several studies reported that the prevalence of COVID-19 pneumonia ranged from 15.7% to 27.1% of patients who tested positive for SARS-CoV-2 [3,4]. Clinical manifestations of COVID-19 most commonly include fever and symptoms such as nonproductive cough and dyspnea, and less frequently headache, myalgia, expectoration, anosmia, ageusia and malaise [5]. However, an estimated percentage of patients ranging from 15.6% to 51.4% are asymptomatic [6,7], and a proportion of them might show characteristic imaging features suggestive for lung damage secondary to COVID-19 pneumonia [8].

The gold standard for the diagnosis of SARS-CoV-2 infection is the detection of viral nucleic acid by reverse transcriptase-polymerase chain reaction (RT-PCR) molecular test performed on nasopharyngeal and oropharyngeal swab specimens [9]. Nevertheless, this test shows a percentage of false negatives estimated to be at least 2% [10]; therefore, medical imaging techniques play a pivotal role in the diagnosis of COVID-19 pneumonia [11]. Computed tomography (CT) is the imaging technique of choice for the diagnosis of COVID-19 pneumonia [12]. Since CT employs ionizing radiation, some authors have suggested magnetic resonance imaging (MRI) as a viable alternative in selected patients [13]. In this report, we present the case of a patient who underwent a contrast-enhanced (CE) MRI of the abdomen which incidentally demonstrated findings suggestive of COVID-19 pneumonia.

## Case presentation

**Figure 1:**
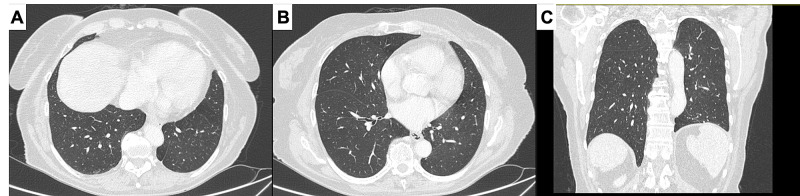
Computed tomography scan of the thorax on axial (A, B) and coronal (C) planes. No ground-glass opacities to suggest novel coronavirus pneumonia nor nodules or secondary lesions involving lung inferior lobes were found.

A 71-year-old woman referred to our department to undergo a CE-MRI of the abdomen due to her oncologic follow-up. She was previously diagnosed with T4a colorectal cancer, treated in 2015 with left hemicolectomy and adjuvant chemotherapy. In 2017, she showed signs of hepatic metastases, with an increase in the number and size of the secondary lesions in the successive follow-ups. She underwent a total body CT scan three months before the study data, which showed an increase in number and size of the liver metastases, along with dilatation of the common bile duct; noticeably, this CT did not demonstrate any lung abnormalities (Figure 1).

The patient was asymptomatic at the time of the MRI scan and had passed the triage done prior to performing diagnostic tests during the second phase of the Italian COVID-19 pandemic (October 2020). The triage checked for the presence of fever above 37.5 °C and symptoms related to infection as well as possible contact with patients with positive SARS-CoV-2 molecular swab specimens in the 14 days prior to the MR examination. Only patients with all negative responses could have diagnostic tests performed following the normal diagnostic pathways in our hospital.

MRI scan was performed with a 1.5T scanner (Siemens Aera, Siemens Healthcare, Erlangen, Germany) using a gadolinium-based contrast medium (Gadoteric Acid). The acquisition protocol included T2 echo-planar fast spin-echo sequences with and without fat tissue signal saturation, diffusion-weighted imaging with apparent diffusion coefficient maps, in-phase and out-of-phase T1 weighted sequences, 3D volumetric interpolated breath-hold T1 weighted sequence with fat tissue signal saturation before and after contrast administration. The acquisitions were performed on multiple planes.

**Figure 2:**
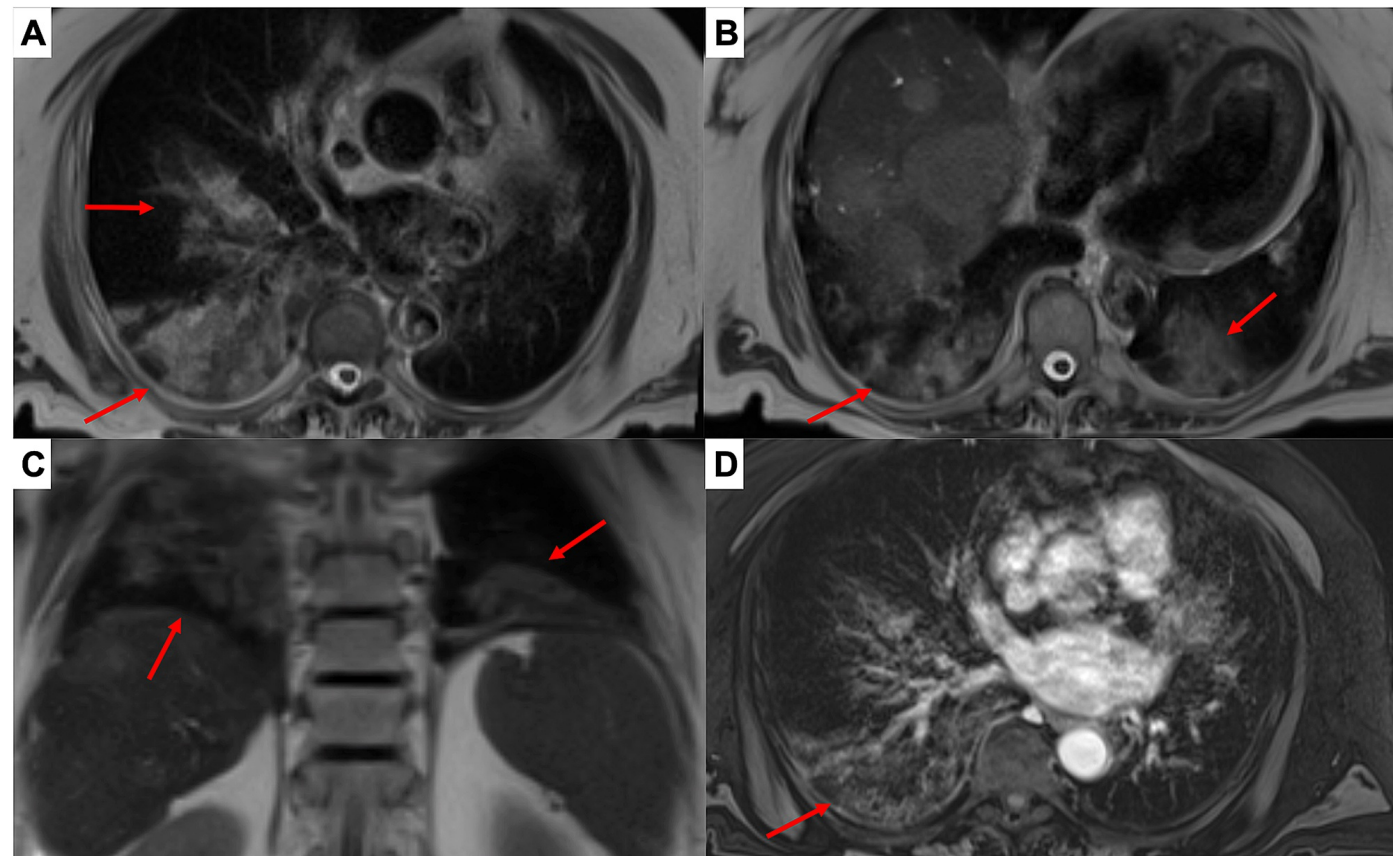
Magnetic resonance of the upper abdomen with a T2-weighted image on axial (A, B) and coronal (C) planes and T1-weighted 3D post-contrast images on axial plane (D). Areas of increased intensity with “ground glass” appearance (arrows) and superimposed inter-lobular and intra-lobular septal thickening were detected. The findings described are localized in both inferior lung lobes with a peripheral subpleural distribution.

The MRI scan showed areas of bilateral and asymmetric increased intensity on T1 and T2 weighted images with “ground glass” appearance of MR signal intensity involving both lower lung lobes, with patchy pattern and preferential peripheral subpleural distribution, with no pleural effusion. The findings were consistent with those observed in patients affected by COVID-19 pneumonia in a previous study comparing CT and MR characteristic features (Figure 2) [14].

The patient was sent to our emergency department and underwent a nasopharyngeal and oropharyngeal swab, which confirmed the diagnosis of SARS-CoV-2 infection. Since the patient was asymptomatic and her oxygen saturation level was within the normal range, she was quarantined at home. We did not perform further imaging studies on the patient since there was no need to expose her to further ionizing radiation (chest X-ray and CT).

The patient remained asymptomatic during the quarantine period, being constantly asymptomatic. After 20 days, she repeated the swab test which was negative.

## Discussion

There is a great interest in the detection of SARS-CoV-2 infection in asymptomatic patients, as they can contribute to the viral spread with similar transmission rates as symptomatic patients [15]. There is numerous emerging evidence of patients affected by COVID-19 pneumonia who are asymptomatic or mildly symptomatic at the time of the diagnosis who show imaging features consistent with lung involvement, similarly to the case report we presented [16,17]. A proportion of these patients might possibly show a sudden worsening of their clinical status, resulting in acute respiratory distress syndrome (ARDS) [18]. Therefore, prompt identification of asymptomatic SARS-CoV-2 infection is very important.

Our experience shows that the use of a triage prior to the diagnostic imaging examinations may not identify asymptomatic patients with SARS-CoV-2 infection. In this way, it can be possible to create a cluster of infection spread within the hospital. The typical CT appearance of COVID-19 pneumonia is a subpleural, peripheral and bilateral ground-glass opacity, with or without areas of consolidation and superimposed inter-lobular and intra-lobular septal thickening (defined as “crazy-paving” pattern) [19].

In this case report, we described MRI features consistent with the peculiar CT findings of COVID-19 pneumonia, i.e., increased intensity areas with “ground glass” appearance and a peripheral distribution involving inferior lung lobes. Although the CT scan of the thorax retains an essential role for the radiological diagnosis of COVID-19 pneumonia, some studies [13,20] demonstrate a nearly complete overlap between CT and MRI findings and diagnostic accuracy in COVID-19 pneumonia diagnosis. We believe that the findings we described were consistent with a case of COVID-19 pneumonia and confirmed that MRI can reliably recognize the signs of interstitial pneumonia. This could be helpful in specific clinical settings regarding selected categories of patients in which CT is indicated and there are major biosafety concerns due to ionizing radiation exposure (e.g., pregnant women and children).

## Conclusions

In conclusion, we described peculiar MRI findings of COVID-19 pneumonia in an asymptomatic patient who underwent MRI as her oncological follow-up. Our case is not only a reminder of the severity of the pandemic that is currently putting a strain on our healthcare system, especially in the identification of asymptomatic patients, but it also confirms the role of MRI as a useful imaging technique in the detection of COVID-19 pneumonia.
